# Personal information and public health: Design tensions in sharing and monitoring wellbeing in pregnancy

**DOI:** 10.1016/j.ijhcs.2019.102373

**Published:** 2020-03

**Authors:** Kevin Doherty, Marguerite Barry, José Marcano Belisario, Cecily Morrison, Josip Car, Gavin Doherty

**Affiliations:** a Copenhagen Center for Health Technology, Technical University of Denmark, Denmark; b Department of Primary Care and Public Health, School of Public Health, Imperial College London, United Kingdom; c Centre for Population Health Sciences, Lee Kong Chian School of Medicine, Nanyang Technological University, Singapore; d School of Information and Communication Studies, University College Dublin, Ireland; e Microsoft Research, Cambridge, England, United Kingdom; f School of Health Sciences, University of Surrey, England, United Kingdom; g School of Computer Science and Statistics, Trinity College Dublin, Ireland

**Keywords:** Wellbeing, Mental health, Pregnancy, Self report, Data sharing, Perinatal depression, Midwifery, Engagement, Disclosure

## Abstract

Mobile technologies are valuable tools for the self-report of mental health and wellbeing. These systems pose many unique design challenges which have received considerable attention within HCI, including the engagement of users. However, less attention has been paid to the use of personal devices in public health. Integrating self-reported data within the context of clinical care suggests the need to design interfaces to support data management, sense-making, risk-assessment, feedback and patient-provider relationships. This paper reports on a qualitative design study for the clinical interface of a mobile application for the self-report of psychological wellbeing and depression during pregnancy. We examine the design tensions which arise in managing the expectations and informational needs of pregnant women, midwives, clinical psychologists, GPs and other health professionals with respect to a broad spectrum of wellbeing. We discuss strategies for managing these tensions in the design of technologies required to balance personal information with public health.

## Highlights

• Sharing personal data in public health is very different from the closed-circle of personal data use.
• Sharing data has both pragmatic (time, workload etc.) and psychosocial (confidence, competence, connectedness etc.) implications for care.
• Health professionals must balance care for the individual against the wellbeing of the patient population.
• Women prefer reflective and conversational, rather than directive or transactional, feedback.
• Designers are advised to focus on strategies to support negotiation, navigate uncertainty, and realise a shared practice of wellbeing.

## 1 Introduction

Researchers and designers have long conceived of mobile devices as tools for the good life (Dow Schüll, 2016). For many, the capacity of these systems to capture our experience, both past and present, suggests their potential to shape our futures towards wellbeing. The constant presence of these devices in our daily lives compels us to ask how the data they capture might enable “a better experience of and in the world” (Elsden et al., 2016). Much HCI research has been motivated by the promise of these technologies to facilitate remembering, reflection, self-knowledge, self-care and change (Hollis, Konrad, Whittaker, 2015, van den Hoven, 2014). Efforts to bring about such outcomes through design, however, are often self-contained, focused, for example, on the engagement of users in a personal quest for insight into thoughts, emotions and behaviour.

If self-reported and self-tracked data has something of value to say about our individual wellbeing, then how might such knowledge contribute to the practice of healthcare on a larger scale? Sharing these new digital representations of our selves opens up new means of realising their potential to support wellbeing. However, the integration of personal information within a public health context also heightens our attention to the inherently relational nature of data and challenges our motivations for monitoring ourselves in the first place.

More mobile applications have been developed to support health and wellbeing in pregnancy than for any other medical domain (Tripp et al., 2014). This is a time of intense personal significance, and a public health priority. Within the United Kingdom (UK), perinatal depression (PND) affects up to 15% of women during pregnancy or within one year of giving birth and suicide is the leading cause of maternal mortality (Knight, Tuffnell, Kenyon, Shakespeare, Gray, Kurinczuk, 2016, Patients Association, 2011). Antenatal depression can also affect fetal development and has been identified as an independent risk factor for children’s emotional, cognitive and behavioural development through adolescence (Braithwaite, Murphy, Ramchandani, 2016, Kinsella, Monk, 2009, Pearson, Evans, Kounali, Lewis, Heron, Ramchandani, O’Connor, Stein, 2013, Stewart, 2011).

In the context of the UK’s National Health Service (NHS), screening for depression is currently carried out verbally and using paper-based questionnaires completed in waiting rooms. It is estimated, however, that at least 50% of PND cases go undiagnosed (NHS Improving Quality, 2015, Thurgood, Avery, Williamson, 2009). Making care and support available to women is therefore a complex challenge which requires engaging a diverse patient population, overcoming stigma, supporting disclosure and fostering trust, while also identifying those in distress who might not otherwise seek help.

Mobile devices are promising tools for screening antenatal wellbeing and depression in daily life. However, these technologies are required not only to support processes of self-report and data sharing but also to enable health professionals to make sense of women’s data, facilitate access to care, and possibly even provide remote feedback and support by means of clinical interfaces. These systems therefore imply an entirely new information ecology which the many stakeholders of personal and public health may view and value differently. This can lead to tensions in clinical practice, and conflicting informational needs. By understanding these tensions, we can aid the design of health and wellbeing technologies, in order to support individual wellbeing on a public scale.

This paper examines the integration of personal information within a public health context. We report on a qualitative design study conducted during the development of a mobile application and online interface for antenatal mental health screening. Through a series of design sessions held with mothers, pregnant women, practice and research midwives, clinical psychologists and other health professionals, we elicit users’ perceptions of self-report, data sharing, data and feedback, responses to interface designs and reflections on the implementation and use of self-report applications and clinical interfaces to support mental health in pregnancy. We identify a set of informational uses and tensions which illustrate how stakeholder values and data come into conflict within the public health context. Finally, we discuss strategies for the development of integrated app and dashboard systems to navigate these design tensions.

## 2 Related work

### 2.1 Public health and perinatal wellbeing

The public health approach aims to shift an entire population towards positive mental health, compelled by the epidemiological evidence for doing so (Huppert, 2014). This objective hinges upon effective programs of assessment, intervention and communication between health professionals and those in need.

Effective mental health screening programs enable services to identify those in distress, efficiently distribute resources, and take appropriate action. These practices are facilitated by screening and diagnostic thresholds which permit the clustering of individuals according to ‘cut points’ along a continuum, often representing the point at which a person “is no longer able to function in their everyday life” (Huppert and So, 2013). How public health services operationalise wellbeing is therefore reflected in the design of mental health screening programs and clinical care pathways. Public health services tend towards measures of illbeing (distress, depression and anxiety) rather than wellbeing (flourishing). Supporting mental health on a national scale however, requires both identifying illness (a pathogenic focus) and promoting wellbeing (a salutogenic focus) (Huppert, 2014, Thieme, Wallace, Meyer, Olivier, 2015).

Mental health professionals have long called for the reorganisation of care to support a more proactive approach enabling more timely support and intervention (Kazdin and Blase, 2011). Multidimensional measures of wellbeing in particular, have the potential to shed light on the relationship between flourishing and mental illness, permitting the “science of well-being” to better inform the practice of clinical psychology (Gallagher et al., 2009). This first requires an understanding of “flourishing in its own right,” and not merely as the “absence of mental disorder” (Huppert and So, 2013). At present, however, measures of wellbeing are largely constrained to the domain of research and “resistance to prioritizing positive outcomes remains high in the field of health” (Huppert, 2014).

At the same time, health tracking technologies are shifting the management of wellbeing away from clinics and professionals and into the hands of patients (Levina, 2012, Schüll, 2016). This ‘new public health’ is characterised by patient participation, a conceptual shift which recognises patients as ‘co-creators’ of their health and wellbeing. Research suggests that patients show improved physical and mental health, maintenance of healthy behaviours and adherence to medication, report greater satisfaction, and make fewer healthcare visits when medical professionals are patient-centred, empathetic and involve patients in decision-making (Shah, Marks, 2004, Williams, Frankel, Campbell, Deci, 2000).

*The Perinatal Context*

Pregnancy is a unique context for the study of wellbeing and a time of pivotal significance for population health — bringing a highly diverse, largely healthy and often strongly motivated population group into contact with medical services (Batool et al., 2017). Midwives’ remit encompasses both physical and mental health and attempts to introduce any new technology into this context must therefore engage with both medical and midwifery models of care; “didactic, factual and authoritative,” as well as “empowered” and designed to foster “greater control” among patients (Smith et al., 2017). Prior research points to the need to recognise and validate both women’s and midwives’ perspectives; experiential and clinical knowledge as well as women-centred and fetus-centred care (Gui, Chen, Kou, Pine, Chen, 2018, Smith, Wadley, Daly, Webb, Hughson, Hajek, Parker, Woodward-Kron, Story, 2017).

Midwifery clinics across the UK screen for perinatal depression through conversations with women and the completion of questionnaires on paper in waiting rooms. According to previous research however, almost half of women report never being told about the possibility of mental health problems, less than a fifth report being completely honest with their midwife, and a third report never telling a health professional that they had felt unwell (Boots Family Trust Alliance, 2013). Depression during pregnancy is marked by an unwillingness to seek help at what parents believe should be a happy time (Moore et al., 2016). The broad emotional experience of pregnancy also makes it difficult for women and health professionals to differentiate depressive symptoms from expected mood and somatic changes (Coates et al., 2015). Many of the 18 symptoms of emotional illbeing described by women in a recent survey do not feature in formal diagnostic criteria such as the ICD-10, for example (Boots Family Trust Alliance, 2013). Concerns therefore often go unreported, despite heightened attention to wellbeing.

Overcoming the mental health treatment gap in pregnancy is a public health priority. This entails the honest disclosure of vulnerability, distress and loss — requiring women to share uniquely personal information within a public health context (Andalibi and Forte, 2018).

### 2.2 Personal health tracking technologies

A significant body of HCI research now focuses on the design of technologies for health and wellbeing (Blandford, Gibbs, Newhouse, Perski, Singh, Murray, 2018, Calvo, Peters, 2014, Sanches, Janson, Karpashevich, Nadal, Qu, Daudén Roquet, Umair, Windlin, Doherty, Höök, et al., 2019). Much of this work has been motivated by the near-ubiquitous presence of mobile devices in our daily lives. These systems are typically idiographic in nature, often designed to facilitate self-experimentation through self-motivated mood, exercise, depression and wellbeing tracking (Karkar, Schroeder, Epstein, Pina, Scofield, Fogarty, Kientz, Munson, Vilardaga, Zia, 2017, Lanzola, Ginardi, Mazzanti, Quaglini, 2014). In the research literature to date, mobile devices have been employed to facilitate the self-report of mental health and wellbeing (Servia-Rodríguez et al., 2017), Parkinson’s disease symptoms (Vega et al., 2018), post-traumatic stress disorder (Larsen et al., 2017), asthma (Buonocore et al., 2017), pain (Adams et al., 2018), stress and sleepiness (Paruthi et al., 2017), bipolar disorder (Matthews et al., 2015), depression (Matthews and Doherty, 2011), anxiety (Topham et al., 2015) and more.

These technologies have been seen as a means to support a variety of objectives, including to generate ‘self knowledge through numbers’ (Ayobi et al., 2018), to support past, present and future-oriented self-reflection (Doherty and Doherty, 2018b), to develop reflective thinking skills (Mamykina et al., 2008), to compare actual, ‘normal’ and ‘ideal’ experiences (Ayobi et al., 2018), to “engender feelings of control and empowerment” (Wichers et al., 2011), to ‘help patients help themselves’ (Wichers et al., 2011), to motivate change and support therapeutic ends (Perrez, Reicherts, 1987, Perrez, Reicherts, 1996, Perrez, Schoebi, Wilhelm, 2000), to enable collaboration with health professionals (Wichers et al., 2011), to adjust medication (Gravenhorst et al., 2015), to alert clinicians to patients at risk, to enable a more proactive approach to care (Shiffman et al., 2008), to serve as a “therapist in your pocket” (Shiffman et al., 2008), to predict the progression of disease (Gravenhorst et al., 2015), to support treatment and intervention planning (Ebner-Priemer and Trull, 2009), to make parenthood a “more quantifiable, science-based endeavour” (Johnson, 2014), to allow health care providers to “discipline parents” (Johnson, 2014), and to map the wellbeing of entire populations.

These many possible styles of use each reflect distinct distributions of knowledge and agency, models of care, ontologies of wellbeing and means of realising positive human experience (Elsden et al., 2016). However, the act of sharing personal data within a public health context represents a significant change from the closed-circle of personal data use and draws our attention to these underlying characteristics of health information practice, as increasingly shaped by technology.

*Perinatal technologies*

At this point in time, millions of pregnant women have installed thousands of mobile applications in the hope of supporting a healthy pregnancy and positive start to life. HCI researchers have developed prototype applications for Dutch (Babywijzer), Pakistani (Baby+), and Vietnamese Australian (We-HELP) populations (Sajjad, Shahid, 2016, Smith, Wadley, Daly, Webb, Hughson, Hajek, Parker, Woodward-Kron, Story, 2017, Wierckx, Shahid, Al Mahmud, 2014), deployed SMS-based systems for personalised health information communication in Kenya and Pakistan (Batool, Razaq, Javaid, Fatima, Toyama, 2017, Perrier, Dell, DeRenzi, Anderson, Kinuthia, Unger, John-Stewart, 2015), and conducted qualitative analyses of pregnant women’s motivations for information sharing and support seeking online (Gui, Chen, Kou, Pine, Chen, 2018, Kraschnewski, Chuang, Poole, Peyton, Blubaugh, Pauli, Feher, Reddy, 2014, Peyton, Poole, Reddy, Kraschnewski, Chuang, 2014). Researchers have also explored the design of prototype technologies for health data tracking in pregnancy, including nutrition, hydration, activity, weight and mood (Bloom) (Wenger et al., 2014), nausea and vomiting (Dot-it) (Lee et al., 2016), and physiological data (Nuwa) (Gao et al., 2014). Peyton et al. propose a ‘pregnancy ecology,’ comprising physical, emotional, informational and social supports, to support the design of physical health interventions (Peyton et al., 2014a), which Prabhakar et al. extend to include support needs, sources, and interventions within an Evolving Ecology of Support (Prabhakar et al., 2017).

Mobile devices are widely believed to possess “the potential to revolutionize clinical treatment” (Shiffman et al., 2008) and there has been a call for public services to explicitly consider mobile applications in perinatal care planning (Tripp et al., 2014). These technologies have the capacity to play an important role in overcoming barriers to mental health screening. However, sharing data might also inadvertently create additional anxieties for patients and clinicians, who face new roles as both producers and consumers of data (Meyer et al., 2018). How personal information is employed within the public health context will inevitably shape women’s and midwives’ attitudes towards gathering, sharing and receiving wellbeing data. Understanding the perceptions, expectations and concerns of these stakeholders with respect to data sharing is therefore essential to the design of effective health and wellbeing technologies.

### 2.3 Clinical interfaces for public health

The wealth of self-tracking data now gathered by patients suggests the value of exploring the integration of this new information source within the context of public health. This in turn, however, implies the need to design clinical interfaces to support practices of data management, sense-making, risk-assessment, feedback and patient-provider relationships (Buonocore, Rocchio, Roman, King, Sarrafzadeh, 2017, Kim, Heo, Lee, Ji, Choi, Kim, Lee, Yoo, 2017, Mentis, Komlodi, Schrader, Phipps, Gruber-Baldini, Yarbrough, Shulman, 2017). These ‘dashboard’ interfaces are distinguished from those employed for post-hoc analysis of research data by the need to facilitate continuous sense-making over longitudinal time-frames.

Several HCI researchers have conducted initial explorations of patients’ and clinicians’ perspectives with respect to practices of data collection and collaborative sense-making. Mishra et al. interviewed hospitalized patients and caregivers to understand how they might together track patients’ health and care within this setting, employing the 5 stages of the personal informatics model (preparation, collection, integration, reflection and action) to structure their findings (Mishra et al., 2018). Mentis et al. explored how Parkinson’s disease patients’ and clinicians’ co-interpretation of step-count data comes to blur “the line between the home and clinic” (Mentis et al., 2017). And West et al. examined the conception of the quantified patient in the doctor’s office; presenting clinicians with vignettes of self-logged pulse rate and caffeine intake data as a means of exploring opportunities and bottlenecks in the use of patient-reported data for differential diagnosis and care planning (West et al., 2016).

Others have examined the design of clinical interfaces. The Ohmage platform, for example, was designed to permit clinicians to view, plot and analyse patient-reported data such as paediatric asthma symptoms and triggers, providing clinicians with a “virtual ‘fly on the wall’ view” of the effects of disease on individuals and communities, reducing hospital readmission rates, and enabling more accurate and contextual prediction of asthma attack risks (Buonocore et al., 2017). The DataMD platform was similarly created to support the integration of patient-reported step, sleep, food, stress, weight and blood pressure data into clinical consultations (Kim et al., 2017). The Monarca platform, described as one possible configuration of a ‘Personal Health Technology’ design space, was developed to enable clinicians, patients and family members to share self-reported mental health data—in order to support treatment, strengthen the therapeutic relationship, and reassure patients (Bardram, Frost, 2016, Bardram, Frost, Szántó, Marcu, 2012, Vilaza, Bardram, 2019).

These studies suggest a desire among patients to track their own health and care in conjunction with clinicians, using mobile technology as a means to assert their own voice, collaborate in the collection and interpretation of data, as well as to better understand the reasoning behind care plans, benchmarks and their own patient history (Mishra et al., 2018). Initial explorations suggest that patient-reported data can improve patients’ understanding of their own health and wellbeing, support their capacity to self-manage (Mishra et al., 2018), improve patient-clinician communication (Kim, Heo, Lee, Ji, Choi, Kim, Lee, Yoo, 2017, Mishra, Miller, Haldar, Khelifi, Eschler, Elera, Pollack, Pratt, 2018), enable more productive reflection through care strategies “attuned to the evolving interpretation of the data” (Mishra et al., 2018), facilitate the discovery and refinement of medical hypotheses (West et al., 2016), and that problems of information overload and time constraints may be overcome by well-designed interfaces (Kim et al., 2016).

However, the study of collaborative tracking and sense-making in the context of public health remains at an early stage. Much research continues to frame the challenges of integrating patient-reported data within clinical contexts as technological and pragmatic, articulated in terms of workload and efficiency, information overload and time constraints, rather than attending to the social and ethical implications of interpreting and acting upon data drawn from diverse sources, and in light of complex, formal and informal relationships and workflows (Gravenhorst, Muaremi, Bardram, Grünerbl, Mayora, Wurzer, Frost, Osmani, Arnrich, Lukowicz, et al., 2015, Kim, Heo, Lee, Ji, Choi, Kim, Lee, Yoo, 2017, Spiel, Kayali, Horvath, Penkler, Harrer, Sicart, Hammer, 2018). There remains a significant disconnect between the design and socio-technological critique of these systems.

Design research has less often focused on patients’ and professionals’ perceptions of data, data sharing or their own capacities with respect to this new information flow in the context of public health (Gravenhorst, Muaremi, Bardram, Grünerbl, Mayora, Wurzer, Frost, Osmani, Arnrich, Lukowicz, et al., 2015, Mentis, Komlodi, Schrader, Phipps, Gruber-Baldini, Yarbrough, Shulman, 2017). When asked, clinicians often express concerns that they do not possess appropriate levels of expertise or training to correctly interpret patient-reported data, given a lack of data representation standards, appropriate tools or familiarity with emerging technologies and the wide variety of data they might face (West et al., 2016). Public health implicates multiple stakeholders; patients, caretakers, clinicians, clinics, medical bodies and even insurance companies (Gravenhorst et al., 2015). In this setting, conflict can easily arise, due for example to ‘segregated knowledge,’ which prohibits patients from attaining the same level of understanding of their health data as clinicians (Chung, Dew, Cole, Zia, Fogarty, Kientz, Munson, 2016, Kim, Ji, Lee, Kim, Yoo, Lee, 2016, Mishra, Miller, Haldar, Khelifi, Eschler, Elera, Pollack, Pratt, 2018).

Furthermore, much research to date has focused primarily on the collection and interpretation of behavioural data such as step-counts rather than self-reported experience as related to the study and assessment of mental health (Kim, Heo, Lee, Ji, Choi, Kim, Lee, Yoo, 2017, Mackillop, Hirst, Bartlett, Birks, Clifton, Farmer, Gibson, Kenworthy, Levy, Loerup, et al., 2018, Mackillop, Loerup, Bartlett, Farmer, Gibson, Hirst, Kenworthy, Kevat, Levy, Tarassenko, 2014, Mishra, Miller, Haldar, Khelifi, Eschler, Elera, Pollack, Pratt, 2018). The characteristics of these forms of data differ significantly, and understanding their interpretation by patients and clinicians is essential for the design of tools to support their analysis. Studies show that health data of all kinds is not ‘self-evident,’ but subject to “manipulation and interpretation based on context and experience,” and that “work must be done in order to use the data in practice” (Mentis et al., 2017). The co-construction of knowledge challenges assumptions of objective truth and expertise, the balance of power in-clinic, and existing models of care (Gui, Chen, Kou, Pine, Chen, 2018, Mentis, Komlodi, Schrader, Phipps, Gruber-Baldini, Yarbrough, Shulman, 2017). Health professionals may experience a ‘lack of expertise,’ for example, when data falls “outside the set of markers normally used for diagnosis” or when visualisations do not easily permit determination of what is “normal (uninteresting) or abnormal (possible evidence)” (West et al., 2016). These epistemological gaps suggest the need for frameworks, models of care and expertise to permit interpretation of diverse forms of data (Ayobi, Sonne, Marshall, Cox, 2018, Larsen, Christiansen, Eskelund, 2017) and to bridge disparities between patients and clinicians who may approach analysis differently, possess divergent goals, or value different kinds of information (Mishra et al., 2018).

*Engaging users through feedback*

Engaging users is a major challenge in the design of health and wellbeing technologies. The collection of valid data hinges upon the engagement of users, often over significant periods of time, and studies frequently report a swift decline in reporting practice following “an initial burst of interest” (Cherubini, Oliver, 2009, Christensen, Barrett, Bliss-Moreau, Lebo, Kaschub, 2003, Dunn, Casey, Sheffield, Newcombe, Chang, 2011). Furthermore, many self-report technologies aim to inspire engagement with processes and outcomes other than reporting alone; to “actively engage patients in the process of recovery” and increase patient insight for example (Doherty, Doherty, 2018, Trull, Ebner-Priemer, 2013, Wichers, Simons, Kramer, Hartmann, Lothmann, Myin-Germeys, van Bemmel, Peeters, Delespaul, van Os, 2011).

Many self-report technologies have been designed to maximise data collection, induce compliance, reduce the burden on users, and support data validity (Ebner-Priemer and Trull, 2009). Although important considerations, framing engagement in terms of these challenges tends to lead to self-contained design strategies focused on self-tracking for personal use or data gathering for research purposes, rather than leveraging social connections within a broader eco-system of data use (Niess and Wozniak, 2018). The study and practice of mental health care specifically recognises social connectedness as a powerful intrinsic motivator and determinant of positive outcomes, as exemplified in measures of belonging and relatedness (van Bel et al., 2009), inter-personal awareness (Watts et al., 1996), social support (Rains and Keating, 2011), working alliance (Horvath and Greenberg, 1989), rapport (Tickle-Degnen and Rosenthal, 1990), therapeutic alliance (Bordin, 1979), social presence, trust and empathy (Gerdes et al., 2010). The combination of personal information and public health evokes many such framings.

This has led to an interest in the potential of clinician-led feedback to engage patients in care, and with respect to technology (Ebner-Priemer, Trull, 2009, Kraschnewski, Chuang, Poole, Peyton, Blubaugh, Pauli, Feher, Reddy, 2014, Robbins, Kubiak, 2014, Torous, Hsin, 2018). Few studies however, have explored how such forms of computer-mediated interaction might shape public health in practice. While providing feedback to patients might facilitate self-report, disclosure, care and wellbeing, it might also create bias, inspire negative forms of reflection and further complicate patients’ and health professionals’ relationships (Ramanathan, Alquaddoomi, Falaki, George, Hsieh, Jenkins, Ketcham, Longstaff, Ooms, Selsky, Tangmunarunkit, Estrin, 2012, Scollon, Prieto, Diener, 2009).

*Balancing informational needs*

Pregnant women, midwives and other perinatal health professionals often possess different goals and values with respect to information use — making design to support information sharing as much a goal-balancing as a problem-solving exercise. This requires a clear understanding of conflicting informational needs, such as that supported by a design tensions framework. This approach has been described by Tatar as a process for drawing attention to the need for reflection with respect to the action of design in light of the potential for conflict (Tatar, 2007).

Design tension analysis is considered especially useful when explicit joint discussion between stakeholders is either not fully possible or desirable (Gautam et al., 2018). Previous studies have identified tensions between the need for social support and privacy in health information use (Maitland and Chalmers, 2018), and between individuals’ informational needs and their desire to promote social interaction within online health communities (Nakikj and Mamykina, 2017). While much research from a design tensions perspective tends to focus on identifying tensions rather than potential solutions, this study aims to do both.

## 3 Methodology

### 3.1 Study aims | exploring the design of a clinical interface

The findings described in this paper arise from design research conducted during the development of *BrightSelf*, a system comprising mobile applications (Android & iOS) for the self-report of psychological wellbeing in pregnancy, a clinical interface enabling midwives to view and provide feedback on women’s reported data, and a server for data storage, management and alert-provision. This system was developed for deployment in a public health service during a clinical feasibility study.

Prior publications have described the study protocol, mobile application design, and a randomised controlled trial of a standalone version of the system (Barry, Doherty, Marcano Belisario, Car, Morrison, Doherty, 2017, Doherty, Barry, Marcano-Belisario, Arnaud, Morrison, Car, Doherty, 2018, Doherty, Marcano-Belisario, Cohn, Mastellos, Morrison, Car, Doherty, 2019, Marcano Belisario, Doherty, O’Donoghue, Ramchandani, Majeed, Doherty, Morrison, Car, 2017). We build upon this research by examining the practice of data sharing in routine care, in order to inform the development and ethical deployment of personal and public health technologies in light of an understanding of patients’ and health professionals’ experiences, values, and concerns.

We organised a series of qualitative design sessions with the aim of exploring the opportunities and design challenges of sharing personal information within a public health context. By presenting and evaluating digital and paper prototypes of mobile and online interfaces we were able to elicit a number of key findings to support the identification of design tensions arising from conflicting informational needs. These include perceptions of self-reported patient data, technology design, and women’s and midwives’ roles in care with respect to the practice of data sharing.

This research forms part of an interdisciplinary clinical research study reviewed and approved by the National Research Ethics Service Committee South East Coast (UK). A research ethics submission for this design research protocol was submitted to and approved by the Head of the Department of Primary Care and Public Health and the Joint Research Compliance Office (JRCO) Co-ordinator at the same institution.

### 3.2 The study design

Between April 2016 and August 2017, 22 design sessions were conducted within the London and Cambridge area by an interdisciplinary team of HCI and public health researchers. Women and health professionals were recruited through social media, the distribution of cards and posters, distant acquaintances, midwifery clinics and research institutions. We aimed to recruit women with diverse experiences of pregnancy and wellbeing, who were either currently pregnant or had given birth in the UK within the last 4 years. In total, 38 participants took part in one of five large group design sessions or one of 17 individual sessions. Individual sessions enabled the inclusion of participants unable to travel or who preferred not to discuss personal experiences in a group setting. These sessions reflected the reality of participants’ daily lives. They were held in women’s homes and university meeting rooms, during breaks at the workplaces of retail managers and stock-brokers and in the kitchens and quiet spaces of midwifery clinics. Six individual sessions were conducted using Skype.

The health professionals who participated in these sessions ranged in age from 25 to 60 and had a variety of ethnic backgrounds as well as experience working with pregnant women through practice and research (See Table 1). All participating midwives were female. The women who took part had all received NHS antenatal care, were aged 28 and older, and had a variety of ethnic backgrounds and nationalities. Those expecting were between the 10^th^ and 39^th^ weeks of pregnancy. Five women had previously given birth and seven had experienced at least one miscarriage. Two women (PW6 and PW8) had been previously diagnosed with depression, and one (PW4) with anxiety. None had received specific diagnoses of perinatal depression, all professed ‘good’ to ‘excellent’ abilities with technology, were in stable relationships, and held a university or college degree. More detailed description of participants’ demographic characteristics can be found in previously published work (Doherty et al., 2018).

**Table 1:** Design research participants.

Participant type	No.	Abbrv.
Mothers (non-pregnant women)	3	M
Pregnant Women	8	PW
Psychologists	1	P
Clinical Psychologists	1	CP
Clinical Studies Officers	1	CSO
Child & Adolescent Psychiatrists	1	CAP
General Practitioners	2	GP
Maternal & Child Health, Obstetrics & Midwifery Researchers/Clinicians	6	CL
Midwives	15	MW

Sessions lasted between 1 to 2 hours and were conducted in two parts. The first sought insight into experiences of pregnancy and perinatal care, motivations for the self-report of wellbeing, perceptions of self-report, data sharing, and the design of wellbeing technologies, in order to ground discussion in professional and personal experience. The second part of each session engaged participants in concept development as well as digital and paper prototype evaluation. Both women and health professionals were presented with patient and clinician facing components of the system and shared their expectations and reflections with respect to degrees of access to data, visualisations, layouts, interface design and midwife-led feedback. Through discussion, sketching, prototyping and testing at each stage of development, we sought participants’ perceptions of the use and sharing of personal information within a public health context.

Nineteen hours of audio were recorded, transcribed and subjected to thematic analysis. Analysis was conducted in parallel by two primary authors working both inductively and deductively with respect to key design challenges and issues arising within sessions related to wellbeing, perinatal care, self-report, technology adoption, engagement, and data-sharing. Tatar’s design tensions framework (Tatar, 2007) was then employed to identify conflicts arising within these sessions as well as additional insights and implications for design. This process entailed identifying conflicting positions, illustrating how tensions are manifested, and how different positions are supported within the literature. Finally, we discuss potential solutions for balancing stakeholders’ informational needs through design, noting solutions previously suggested within the literature and the potential for unintended consequences.

## 4 Findings | Sharing information in the clinical context

These sessions explored participants’ impressions of the practice of remotely sharing personal data with midwives, including self-reported mood, sleep, enjoyment, energy, worry and Edinburgh Postnatal Depression Scale (EPDS) scores. The themes which emerged from these design sessions therefore reflect how pregnant women, health professionals and public health services each uniquely interpret the implications of data, what it means to be well, the optimal distribution of resources, communication styles, and the characteristics of the public health context.

### 4.1 Perceptions of data sharing

Women’s responses to the practice of data sharing reflected both enthusiasm and anxiety. One woman (PW6) who had previously suffered from depression spoke of her hope that sharing data would provide her midwife with the opportunity to express care and reassurance, articulating her sense that, in the past, nobody “really show[ed] any interest in...how I feel...nobody asked.” M3 envisioned the use of a mobile application as a means of overcoming cognitive limitations; “I might have forgotten what happened two weeks ago...if they would retrieve it and then say ‘Oh you mentioned this...this thing happened, do you want to share more?’ ” For M2, who had at times been unhappy with her midwives however, the idea of sharing mental health related data was simply ‘bad.’ As PW2 states, “mental health, that’s the one thing maybe that is a bit different. Definitely taboo to say you don’t really like being pregnant much.”

Women’s attitudes towards data sharing were therefore shaped by the questions posed by the technology. The EPDS questionnaire, for example, was perceived as particularly medical in nature and associated with negative, stigmatised and non-negotiable consequences; “I think you’d find it quite hard to be honest about that [the EPDS], if you knew your midwife was seeing it” (PW2), “because the language is quite clinical...I will think twice before replying to it” (PW3), “I don’t want them to think that I’ve got depression, because then that means it would go on my record, it might affect whether they believe I can look after my baby...it would affect my level of honesty I think, in reporting” (PW7). Women also clearly related the value of data sharing to the severity of their own need; “if a person is asking for help...wants some help...it’s really useful. Whereas if a person is doing fine it’s like ‘oh, why are you intruding on my space’” (M3).

Midwives in turn expressed awareness of their need to play a role as gatekeepers to care, and their broad remit with respect to individual women’s physical and psychological needs as well as clinical and organisational structures. Discussion among health professionals often turned to the question of how an additional data source might interfere with their relationships to women; “It’s a little ‘big brother’?” (MW3 | Female), “You’ve got all my information, why are you asking me again?” (CL2 | Female).

### 4.2 Perceptions of the client-midwife relationship

Attitudes to data sharing, and disclosing mental health more generally, were described as highly dependent on the relationship between women and their midwives. PW6, for example, commented that her desire to share her data was contingent upon her midwife appearing ‘sensitive,’ “interested in mental health,” and capable of assessing her data “in a more calm way.” Women spoke of significant variance among midwives. PW7 explained, “you get the ‘earth mother’ types, and then you get the more ‘clinical matron’ types.” Others were sceptical of midwives’ abilities to handle their data. PW4 noted that her diabetes and insulin related data was used “very ineffectively” by her nurse and diabetic obstetrician, observing that “it’s overwhelming for practitioners...[given]...the amount of time that they have.” PW7 was keen to stress her perception that midwives are not “data driven professionals, like you might get clinicians or consultants are, or scientists...the midwives I’ve worked with, they are...um...they’re not really on the ball with this sort of stuff.”

Other women shared the impression that “a midwife is not a mental health professional” (PW8). For PW3, sharing data related to her mental health would prove valuable only if her midwife “has received training, and when I’m talking about training, I’m talking about therapeutic training, about how to handle with care the data.” Both women and midwives highlighted the power-dynamics implicit in data sharing; “she knew so many things about me, I didn’t want to share everything [emphasis] with her” (M2). PW3 was keen to avoid a mode of interaction driven by scores and thresholds, “You scored 10 out of 10, good one!’ I don’t want to have this kind of chat with my midwife.” Midwives, meanwhile, were also keen to avoid presenting themselves as mental health professionals or increasing women’s distress; “people who are anxious are very focused on threat, so they’re much more likely to...interpret something as threatening and to feel even more anxious because of that” (CL2 | Female).

### 4.3 Perceptions of data and its implications

Discussion of technologies to support self-report, data sharing, and sense-making is often shaped by perceptions of ‘data’ itself and assumptions concerning its meanings in a public health context. Face-to-face meetings, phone calls, scribbled notes, mobile applications and questionnaires completed on paper are all means of sharing information employed by women and midwives. ‘Digital data,’ however, carries an additional set of meanings including the assumption of a more rigid form of truth which necessitates supplementary actions and responsibilities.

Participants often spoke of the implications of ‘data.’ Data might have a refocusing effect for example, causing midwives to become “more focused on my self-reports as opposed to maybe signs that she should notice...if she notices me sobbing for something silly, then that’s her cue that ‘maybe I should ask her about her mental health’ ” (M2). Women spoke of conflicting needs for accuracy and privacy related to the potential interpretation, and over-interpretation, of their data;

*“Let’s say I’m upset today because my son upset his room, for a very stupid reason, ok, and I write ‘I’m upset today,’ and the next day, for example, I have a fight with my husband, and the third day, I have a fight with my friend, and the fourth day, whatever, I got a really high electric bill, ok, so this looks like a really bad week, and then the midwife, when I meet her,...the midwife might say ‘Oh well look, there’s a trend here.’ Maybe there isn’t a trend [emphasis].” (M2)*

Women also raised concerns, however, with respect to the sharing and interpretation of open-ended or fine-grained data which might reflect causes of distress unrelated to pregnancy, and which fell outside the remit they were comfortable granting to midwives. Sharing extensive wellbeing data could impose an onus on women to explain the reports they provide, leading to unwanted intrusion into their lives, “Honestly, if I told my midwife ‘Oh, I had a fight with my husband’ and then the midwife had a comment on marital relationships or my husband...[laughter]” (M2).

Meanwhile, the terms ‘objective’ and ‘subjective’ often featured in participants’ reflections. Quantitative data, such as blood pressure, sugar, weight and EPDS scores, were often considered more fixed, factual, measurable and objective in nature. Two midwives discussing this point, for example, suggested that face-to-face contact was ‘subjective’ whereas a prototype clinical interface was more ‘objective’ (MW13 | Female & MW14 | Female). Such interpretation suggests the potential to over-attribute validity to digital data as well as under-attribute meaning to disclosures made face-to-face. This risk is potentially particularly high when data reflects inherently subjective phenomena such as self-reported symptoms of mental health which are subject to a variety of biases; “It can be skewed you know. Only when you’re desperate and feeling really lousy then you go on it” (M3). As GP2 states, the act of gathering data pertaining to mental health in a clinical context “has a whole load of other problems and worries about involving social services and children being taken away and all that sort of stuff. It’s not, I don’t think it’s the same as black and white physical data.”

Health professionals also tended to attribute significant exigency to data. Midwives in particular, exhibited a preference for technologies which allowed them to use ‘their area of expertise’ and to collect information they could ‘act on.’ Perhaps paradoxically however, the significant and ‘objective’ onus for action which some midwives associated with digital data meant that it risked becoming a source of anxiety. Professionals expressed a need to balance the information sought from women against their ability to take appropriate action based on those disclosures; “I can’t look at every woman that, you know...there’s six thousand women, potentially, that’s a thousand women, 20% of women could be low, so that’s an awful lot of women” (MW11 | Female).

Women were keenly aware of the pressure on midwives and described this as a factor in their willingness to disclose their emotional experience in face-to-face meetings. Participants also stressed, however, that they did not want technology to replace “that personalised touch,” becoming an “avenue for the midwife to cut short the interaction with a patient,” which “defeats the purpose” (M3). Interestingly however, other forms of technology were not seen as subject to the same challenges attributed to self-reported data. All participants frequently spoke of the routine use of phone calls, text messaging and email; “really happy to talk face to face or at the end of a phone” (MW5 | Female). PW6 noted that text-based communication was now simply a normal means of interaction; “everybody now is communicating with messages so...it’s very common...this is how we communicate.” Women often referred to midwives’ informal use of their patient records, which women themselves carry between appointments, to communicate important information within the team; “she wrote all of that in my notes, and I guess maybe that would have been helpful for the staff when I was giving birth, to know where my family was” (M2).

### 4.4 Perceptions of wellbeing and the challenges of assessment

Midwives often stressed the challenges entailed in obtaining an accurate perception of women’s wellbeing, stating for example that many women “don’t actually even share some of their mental health issues with their next of kin” (MW10 | Female). They praised a version of the Whooley questions which includes the question “do you want help with anything?...which I think is useful in the sense that it asks women, it empowers women so that you are actually asking them do they want help with anything rather than suggesting that they need help. So it’s up to them to identify” (MW7 | Female).

When it comes to gathering data spanning a spectrum of wellbeing from measures of depression to flourishing, clinicians expressed a desire for a more familiar means of interpreting such data, similar to blood tests for example, “they just pull them up on a screen, they get, ehh, this is a score, is it inside or outside a range and they act upon it” (CL6 | Male) or child protection; “a red alert or some warning then we know...look at those women when it comes up...[speaker change] yeah that’s a good idea?” (MW14 | Female). Discussions of ‘data’ therefore often spoke to a broader set of concerns pertaining to the assessment of wellbeing on a population scale. Clinicians commented that face-to-face interaction provides more frequent opportunities to employ their own judgement, allowing for pragmatic concerns, including pressure on resources, to be considered. In this way, the threshold of what constitutes illbeing is determined more informally than that articulated by a screening questionnaire such as the EPDS. Alternatively, however, this may also provide a means of avoiding certain problems whose expression could increase clinicians’ workload and anxiety, or create significant organisational challenges. As one female GP (GP2) notes “sometimes people don’t want to pick up problems that might take more time.”

### 4.5 Perceptions of dialogue and feedback

We also explored women’s and midwives’ attitudes towards midwife-led feedback provided through a mobile application. Many women felt that some form of feedback would “show that the midwife cares, because at the moment it doesn’t feel like there’s much time for interaction” (PW1), “it does feel like you’re a bit on your own because we don’t have that many touch points” (PW2), as well as provide reassurance; “I would like some kind of feedback, as in ‘that looks serious, we’re going to look at this’ and you’ll get a phone call, you’ll get an email, you’ll get something” (PW4). Women also suggested that feedback could motivate their engagement in self-report, “oh, somebody is looking at you, you feel a connection to the app...and maybe mothers would be more willing to use it” (M3), as well as provide other advantages, “maybe it’s a discreet way to, actually to, if a woman is abused or whatever, it’s a way to send her [a] message” (PW6). Although health professionals were keen to stress the burden on midwives, “some nights you just don’t even get time for a break, so you have to make it as simple as possible for the clinicians” (CL2 | Female), they also spoke positively of the value of, and even need for, feedback if patient-reported data was to be shared with clinicians; “you need to say we’ve received your data, it’s ok, it’s not ok, so there’s got to be some type of feedback” (CL6 | Male), “it’s not just gone out into cyberspace” (CL2 | Female).

Opinions tended to diverge however with respect to the nature of that feedback. Several women spoke positively of feedback which referred directly to the data provided, “that could be useful, if a midwife notices that there’s a very negative mood, and then tells you ‘hey, you could do stuff like take some me time’ ” (M2), “something positive if somebody’s doing well...or if somebody’s worried...ask if he [sic] needs help” (PW6), or “some other tip of the day that sounds like it might be coming from a midwife” (PW8). Midwives, however, voiced caution around labelling an emotional state based on data provided through an app; “they might think ‘well hold on a minute, I don’t feel that’ ” (MW7 | Female), “I wouldn’t want to say ‘come and see us! [laughter] we’re worried about you!’ ” (MW5 | Female). This cautionary tone applied also to feedback itself; “because...what we think as positive might not be seen as positive at the other end” (MW4 | Female), “if you reinforce ‘you’re feeling low in mood...’ they might just take that coat and wear it, but if you say ‘you’re not feeling your normal self,’ that’s just fact isn’t it?” (MW11 | Female). PW3 shared these concerns; “I think we should avoid the creepy situation of sending a self-assessment and having someone coming back to you with...a feedback which is really precise.”

Instead, midwives expressed a preference for simpler forms of messaging from view notifications to appointment reminders or recommendations for events such as parent evenings; “signposting about events and things like that is...[a] much...safer bet” (MW4 | Female). Several women also expressed support for such feedback; “I think the midwife’s job could be to...add a little message that says ‘Oh there’s a...mindfulness group or zumba group for pregnant ladies or whatever coming up’ ” (PW8). PW4 suggested that even the simplest forms of communication might support a more positive working relationship.

Regardless of the specific form of feedback, both women and clinicians stressed the importance of an appropriate tone. Health professionals suggested that feedback might function best if phrased as an offer of support; “would you like to speak to someone?” (MW1 | Female). Women voiced a preference for reflective rather than directive, and conversational rather than transactional, feedback; “‘you appear to be feeling quite sad,’ or ‘you’re not sleeping very well at the moment, is this something that you’d like help with?’ which feels very different to ‘you’re 200% over the norm!’ ” (CL3 | Female).

Questions of time and timings were also raised during discussion of feedback. PW1 expressed a desire for an appropriate delay before any feedback of a direct nature; “I think if I had reported, if I’d gone on there and shown that the whole week I’d felt pretty bad and I’d typed that in constantly, I would feel comfortable someone contacting me because...that would show that that’s a fairly prolonged period.” PW5 considered routine important; “I think it might be more reassuring if it was going to be the case that your healthcare professional was gonna kind of feedback on it regardless.” PW2 called for feedback to be aligned with the practice of antenatal care; “feedback like bi-monthly, I think just to have that touch-point in between [appointments], that would be quite...good.” Finally, PW6 suggested that any protocol would work best as the result of a negotiation between a woman and her midwife; “When you have your first appointment and you introduce this...the patient and the midwife can discuss what expectations are and eh, how often he [sic] wants to communicate, or you know, they can say we can check this on a week basis, and they can agree on that. I don’t think it’s such a problem.”

### 4.6 Public health in society

*“I think if you could start people talking sooner about things that they’re finding challenging about impending motherhood, then maybe it wouldn’t be so hard for people to talk about things they’re finding challenging about actual motherhood once it arrives.” (PW5)*

Although these sessions focused on the act of sharing data within a clinical context, this did not preclude comments which reflected the broader social context of pregnancy and the situated reality of women’s lives. One woman remarked that, “it’s pretty rare that you get presented with the genuine full spectrum of things that you might be feeling, in a way which is compelling” (PW5). Midwives often raised pragmatic issues of workload, discontinuities in care, and their role in the distribution of resources but also reflected the need to balance care for the individual against the wellbeing of the patient population. Midwives stated that this was “really difficult” due to a case-load “so varied that you don’t necessarily get that continuity” (MW3 | Female) and therefore, although “women...are generally using apps more and more in their daily lives so I think it does certainly enhance and benefit them,” “we don’t have the, um, ability or access to use apps in our work, never mind the space to get onto the computer” (MW8 | Female). Clinicians stressed the need for any new technology to be “integrated with what systems people are using” (CL3 | Female).

The question of patient empowerment within organisational structures was raised by midwives who asked, “will you be wanting more women to present or wanting them to feel more empowered about their own mental health and take control?” (MW8 | Female). One midwife (MW11 | Female) openly expressed a fear that, by rendering the needs of a population more starkly visible and collating patients’ data in one place, a patient-reported data system might evoke the sense that “oh my god I’ve got to bring in all these women because their mood is very low.”

## 5 Discussion | Tensions and strategies

These themes therefore reflect a wide variety of aspirations and concerns with respect to the design of health and wellbeing technologies. We next identify and discuss three core tensions which featured throughout these sessions, and suggest strategies for their negotiation through design in light of the need to balance personal information with public health (See Table 2).

**Table 2:** Tensions and strategies.

Tensions	Strategies
Sharing vs. Monitoring	• Recognise the Co-Creation of Health and Wellbeing within a Broad Spectrum of Data • Provide Effective and Practical Feedback • Prioritise the Midwife-Client Relationship
More and Less Data vs. True and False Confidence	• Design to Navigate Uncertainty • Enable Clarity of Sense-Making to Support the Practice of Care • Acknowledge Subjectivity by Connecting Data to Goals and Values
Personal Information vs. Public Health	• Attend to the Framing of Personal Experience within Public Norms • Attend to the Propagation of Epistemic Values • Support Transparency to Mitigate Misinterpretation • Acknowledge Personal and Public Framings of Wellbeing

### 5.1 Sharing vs. monitoring

*“It’s the way you ask a question sometimes that brings about a useful answer.” (MW11 | Female)*

Effective mental health screening programs are required to balance the sensitive presentation of avenues for disclosure with the need to survey populations and pro-actively identify those at risk who might not otherwise feel able, sufficiently self-aware, or willing to seek support. Clinical self-report technologies are therefore required to navigate an intrinsic public health tension: the ‘sharing-monitoring dilemma.’ How can a public health service facilitate support-seeking behaviours while also identifying those at risk through practices of assessment?

These design sessions demonstrate that activities of sharing and monitoring are far from easy to reconcile, entailing both compassionate and deterministic framings of distress as well as unequal distributions of power and autonomy. Women often spoke of the extent to which midwives’ capabilities, practices and attitudes shaped their willingness to disclose their own needs, wellbeing and levels of distress. This suggests that the more midwives’ activities are perceived as motivated by assessment, the less likely they are to facilitate honest disclosure, raising significant challenges for design.

Perceptions of monitoring and sharing are not fixed from the outset but derive from the accumulated weight of numerous actions and choices. This can affect which questions are asked and under what conditions, or which data is shared when, and with whom. Each of these decisions has implications for who makes sense of this information and how it will be acted upon. These questions apply as much to face-to-face conversations as to data shared through a mobile application. In digital interactions, as in face-to-face settings, these tensions are therefore not to be resolved but attended to; negotiated in practice and by design.

Midwives view the introduction of new sources of data as a potential burden upon already stressed services. Their need to distribute resources and elicit actionable forms of disclosure can clash with a woman’s need to make sense of her own experience. However, some women suggest that these practices might also permit clinicians to share the ‘workload’ and responsibility for patients’ health with patients themselves. In doing so, this enables a variety of design strategies to support negotiation:

#### Strategy 1. Recognise the Co-Creation of Health and Wellbeing within a Broad Spectrum of Data

The negotiation of care is made possible by recognising that clients and health services are co-creators of their health and wellbeing. This conceptual framing provides the context for a relationship which allows women to be treated more fully as actors (subjects) rather than sources of data (objects) to be monitored, and for conflicting needs to be addressed. This in turn requires shared access to informational content that enables dialogue. These sessions revealed how strongly perceptions of data shape practice — often towards rigid, constrained and deterministic interpretations. This suggests the importance of a second distinction, orthogonal to concerns of sharing and monitoring, between pathogenic (identifying distress) and salutogenic (understanding wellbeing) measures of wellbeing.

Employing a broader spectrum of wellbeing data, and making sense of its informational content in collaboration with patients, may challenge clinicians’ perceptions of the ‘objectivity’ of subjective experience. Recognising the social and ethical character of data collection also facilitates new forms of interpretation and design; including whether data needs to formally dictate interactions in-clinic for example, or might be employed to engage patients in more supportive conversations (Larsen et al., 2017). Less transactional forms of interaction design can provide opportunities for negotiation and increase patients’ willingness to disclose their emotional experience.

A fuller understanding of the spectrum of emotion and wellbeing in pregnancy may also enable more effective programs of assessment. This could facilitate more timely identification of depression and depressive symptoms, making treatment and support available to those in need. Designers of health and wellbeing technologies can therefore mitigate unhelpful perceptions of assessment and monitoring, driven by narrow conceptions of wellbeing, by incorporating a broader selection of data for diverse purposes including the creation of narratives of care which better encapsulate the shared aims of personal and public health.

#### Strategy 2. Provide Effective and Practical Feedback

Participants’ comments suggest that clinician-led and technologically mediated feedback can provide value to users as well as an incentive for repeated engagement (Dunn, Casey, Sheffield, Newcombe, Chang, 2011, Santangelo, Ebner-Priemer, Trull, 2013). Attending to the provision of feedback may also allow designers to move towards narratives of reciprocity rather than compliance, and therefore shape a context in which to support shared decision making, the negotiation of care, and disclosure.

Feedback is a complex process which includes features related to the method of delivery and display (notification design) (Muench et al., 2013), motivations for feedback (self-awareness, behaviour change, coping and conversation) (Muench et al., 2013), persuasive strategies (Heron, Smyth, 2010, Kazdin, Blase, 2011, Ryan, Deci, 2008), message content (Heron and Smyth, 2010), communication media, tone and phrasing (Batool, Razaq, Javaid, Fatima, Toyama, 2017, Kazdin, Blase, 2011, Muench, Weiss, Kuerbis, Morgenstern, 2013), timing (Hareva, 2009, Heron, Smyth, 2010, Muench, Weiss, Kuerbis, Morgenstern, 2013, Parks, Schueller, Tasimi, 2012, Schueller, 2010), and the conflicting needs of clients and health professionals (Muench et al., 2013). Initial studies combining technological feedback with therapeutic techniques have found positive effects (Heron and Smyth, 2010). However, the design, practice and effects of feedback remain understudied and significant further research is required to develop our understanding of the development of appropriate protocols (Donker, Petrie, Proudfoot, Clarke, Birch, Christensen, 2013, Dunn, Casey, Sheffield, Newcombe, Chang, 2011, Gentles, Lokker, McKibbon, 2010, König, Aalten, Verhey, Bensadoun, Petit, Robert, David, 2014, Preschl, Wagner, Forstmeier, Maercker, 2011).

**Fig. 1:**
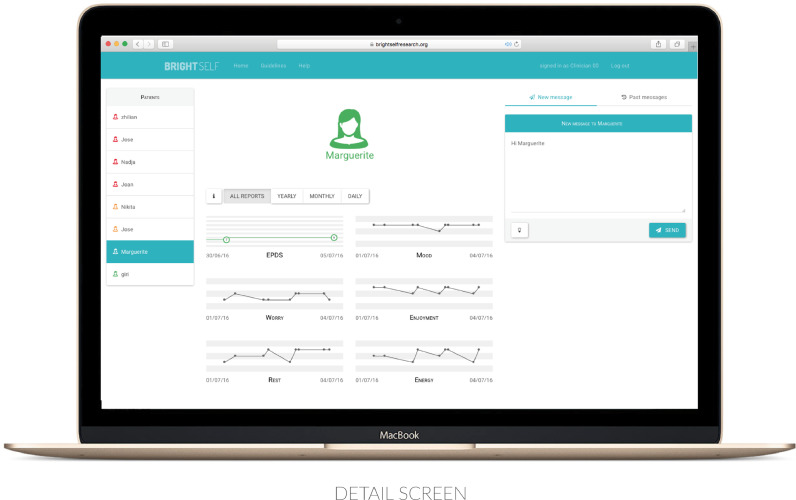
The detail screen.

These sessions highlighted the need to reduce the burden on those supplying feedback by providing efficient summarisations and visualisation of women’s data. This approach informed the development of a clinical interface to facilitate future practice and research efforts by enabling the delivery of midwife-led feedback to a client’s personal device. These insights also motivated the design of a set of five personalised message templates (data acknowledgement, appointment reminder, event suggestion, general advice, and data-driven feedback) for tailoring by midwives, in order to reduce the workload on clinicians and provide guidance in the practice of feedback (See Fig 1). A decision was made to support only one-way communication of this kind, in order to reduce the potential for anxiety stemming from the need to respond, and to maintain the priority of the clinical visit.

#### Strategy 3. Prioritise the Midwife-Client Relationship

While feedback might also be provided by automated systems, yielding economic and human workload savings (Hareva, 2009, Heron, Smyth, 2010, Trull, Ebner-Priemer, 2013), these sessions also highlighted the need to recognise the value of the midwife-client relationship. Supportive relationships foster wellbeing and shape positive perceptions of monitoring, connectedness, care and support. They may also play an important role in supporting the adoption of healthcare technologies (Webb et al., 2018). Data can be gathered and shared not only for its informational content but to provide reassurance, share ownership of the responsibility for patients’ health and wellbeing, and strengthen the bonds between pregnant women and health professionals. Participants’ comments encourage us to consider mental health technologies as component parts of an ecology of care whose efficacy hinges in large part upon perceptions of trust, empathy and interconnectedness. Torous and Hsin have recently spoken in similar terms of the need to conceive of mobile health applications not only as ‘discrete tools’ but as means to strengthen and support ‘digital therapeutic relationships’ (Torous and Hsin, 2018).

Clinical mental health technologies can support positive relationships by highlighting the range of possible motivations for data sharing as well as adopting a sensitive tone in light of the vulnerability entailed in the disclosure of mental illness. Designers can also seek to support flexibility, trust, authenticity, empathy, understanding and accountability by setting appropriate limits on communication, and focusing on strategies to support social connectedness, support, and relationship-centred care (Aguilera, Muench, 2012, van Bel, Smolders, IJsselsteijn, de Kort, 2009, Borrell-Carrió, Suchman, Epstein, 2004, Cohen, Wills, 1985). Mental health technologies might, for example, embody the characteristics of effective therapeutic relationships, including exploration, reflection, flexibility, interest, warmth and openness, noting past therapeutic success and accurate interpretation, facilitating the expression of affect, and attending to the patient’s experience (Ackerman and Hilsenroth, 2003).

### 5.2 More and less data vs. true and false confidence

These sessions reveal the extent to which introducing a new source of knowledge into the perinatal context requires attending not only to the rational interpretation of that data but the psychosocial implications of its interpretation by women and healthcare professionals. Much of the promise of technologies for self-report and data-sharing hinges upon their potential to improve the ecological and temporal validity of the data available to patients and health professionals, supporting increasingly evidence-based practice. However, increased access to knowledge without the means or skills to interpret it risks undermining midwives’ confidence in their abilities and women’s trust in midwifery services. During these sessions, both women and clinicians expressed fears around midwives’ capacities to cope with new sources of data.

Health professionals often voice concerns that digitalisation will threaten their professional autonomy (West et al., 2016). Writing a patient’s commentary down changes its significance, its potential, its potency. And, with a clearer record of events comes greater scrutiny, potentially stifling opportunities for disclosure and narrowing options for care. Studies of clinical decision making indicate that “confidence is valued over uncertainty” and that there often exists “a prevailing censure against disclosing uncertainty to patients” (Croskerry, Norman, 2008, Kahneman, 2011). On the other hand, restricting data gathering efforts to a smaller set of ‘objective’ measures in order to support increased confidence, and perhaps competence, also has the potential to realise a narrower form of understanding which, in its own turn, stifles options for care.

Participants displayed a tendency to associate objectivity and rationality with digital data. Indeed, tracking our health and wellbeing entails bracketing our felt experience, and the clinical interfaces of the future may render our selves, in the form of patients, as bits and pixels. This association echoes perspectives from science, technology and society studies (STS) which suggest that wearable tracking devices invite us “to view ourselves as longitudinal databases constantly accruing new content: ‘You are your data’ ” (Schüll, 2016), risk reducing the self to endlessly divisible data points, not individuals but “dividuals and masses, samples, data, markets, or banks” (Deleuze, 1992), or that these “healthy lives are re-ontologised lives” (Spiel et al., 2018), defined in terms of the data available to us.

Clinicians’ perceptions of the objectivity of digital and numerical data may not represent a purely epistemic stance but a means of narrowing the bounds of ambiguity and granting the confidence required to take action in the face of uncertainty. For health professionals, working under ‘extreme uncertainty’ is often a necessity which must ‘be embraced’ (West et al., 2016). Making sense of this uncertainty requires knowledge, expertise, and now also digital literacy. As Elsden et al. note, although “complexity, uncertainty, and ambiguity may run counter to self-knowledge and belief in technological deliverance,” these are “fundamental elements of the human experience that deserve recognition and consideration in design” (Elsden et al., 2016). Midwives do not see themselves as mental health or data professionals but interpret activities of clinical data gathering and sharing in light of a ‘best practice’ confidence interval amenable to design. Enlisting the following design strategies may therefore grant clinicians the capacity to navigate competing concerns of confidence and competence;

*Strategy 1. Design to Navigate Uncertainty*

We can begin to embrace uncertainty by recognising where it already exists in clinical practice. This requires an honest engagement with professionals’ capacities and expertise, recognising a reluctance to admit to ambiguity in practice. This also means engendering trust in interpretation and clearly expressing the limits of that knowledge — making clear when data can and cannot be trusted. The *BrightSelf* interface was designed to support clarity of interpretation by means of a shallow learning curve, clear and minimal navigation, descriptions of scales and thresholds, colour-coding of data visualisations according to severity, and layouts to enable efficient comparisons between data of different forms. Designers might also employ such strategies as choosing to avoid free-text responses in light of the challenges associated with interpreting such data or responding with appropriate urgency.

*Strategy 2. Enable Clarity of Sense-Making to Support the Practice of Care*

**Fig. 2:**
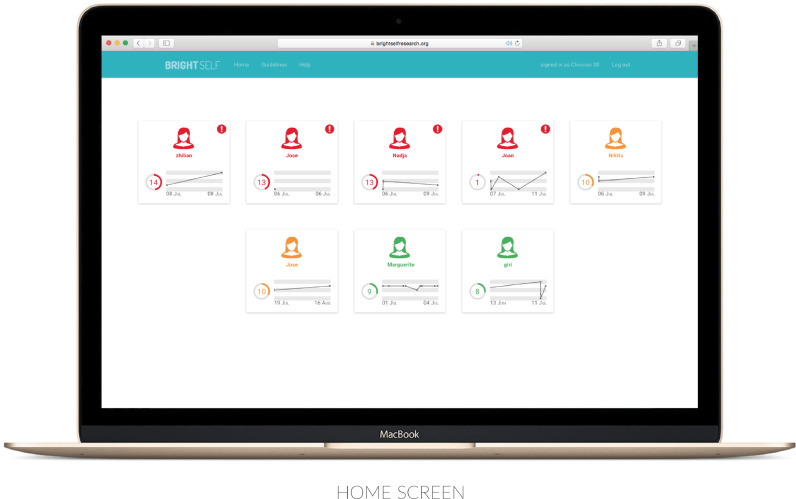
The home screen.

Prior research consistently describes pragmatic concerns of time, workload and the continuity of care as among the primary determinants of the successful adoption of clinical healthcare technologies (Doherty, Coyle, Matthews, 2010, Doherty, Barry, Marcano-Belisario, Arnaud, Morrison, Car, Doherty, 2018, Gentles, Lokker, McKibbon, 2010, Webb, Wadley, Sanci, 2018). During these sessions, many participants also commented on the extent to which pressure on time and resources shapes the experience, and practice, of care. Midwives often spoke of challenges pertaining to sense-making and action: “we know ourselves that when women make a referral, sometimes we have to chase it up so many times, just to make somebody urgently seen” (MW10 | Female). It is often reported that mental health professionals experience difficulty “interacting with participants without any progress data” (Prochaska and Velicer, 1997), and learning from clinical experiences when they do not receive accurate feedback, or “when their cognitive processes are inadequate (i.e., when they remember information incorrectly)” (Garb, 2000). Self-reported data may therefore enable midwives to develop a more accurate picture of their clients’ wellbeing, while facilitating digital practices of triage and referral (See Fig 2).

*Strategy 3. Acknowledge Subjectivity by Connecting Data to Goals and Values*

Concerns of confidence and comprehension are closely related to the types of data with which we engage. Patient reports of subjective experience, for example, are more evidently subject to interpretation than step counts. As Hollis et al. write, in contrast with ‘automatic logging,’ “active, conscious reflection on events may be an important facilitator of behaviour change” (Hollis et al., 2015). Niess et al. in turn suggest that the use of fitness trackers is directly related to users’ need for an explicit connection between the numerical values presented by tracking technologies and their personal fitness goals (Niess and Wozniak, 2018). These sessions also highlighted the need to mitigate the over or under-attribution of meaning to data in clinical contexts. Designers are therefore advised to strive to support clarity of presentation and interpretation by acknowledging where uncertainty arises from subjectivity, by experimenting with narrative rather than purely numerical framings of knowledge and feedback, and by seeking to support meaningful connections between users’ data, goals and personal values.

### 5.3 Personal information vs. public health

*“A lot of women who have postnatal depression or anxiety,...they know they’re struggling but they’re not sure if everybody, if that’s normal” (CL2 | Female)*

Surrendering our personal data to a public system opens it up to new uses and meanings. The final tension highlighted by these sessions reflects the relationship between self and other as expressed through the need to know ‘what’s normal’ during pregnancy. We gather data to support self-knowledge and personal wellbeing yet are compelled to turn outwards, seeking a frame of reference with which to make sense of our own experience. Thus, the act of data sharing recasts our motivations for gathering data in the first place.

While data can serve as a means of expression, communication and access to care, it can also introduce additional pressures and responsibilities. These representations of our selves permit new forms of knowledge and action. They also reflect ‘what matters’ to us and are at times braced between our desire to exert control over our self-presentation and our resistance to the collective imposition of an obligation to do so. This is a particularly acute experience for pregnant women, whose autonomy is collectivised through public health services and placed in balance with that of (and responsibility for) the foetus (Verbeek, 2008). A mobile app designed to “responsibilise parents for their children’s health monitoring and developmental requirements” can also allow clinicians to “discipline parents who have missed developmental health checks such as immunisations,” serving “as a tool for surveillance and discipline as well as convenience” (Johnson, 2014).

These design sessions often reflected normative concerns, whether with respect to mental-health related stigma, the need to know what’s ‘normal,’ or organisational requirements. Many self-tracking technologies aim to uncover how we ‘should’ live through apt description of our lives. Designed to infer ‘ought’ from ‘is,’ these systems therefore defy Hume’s fact-value dichotomy, the claim that ‘matters of fact’ are entirely separate to ‘relations of ideas,’ including questions of right and wrong, ill and wellbeing (Hume, 1738). Digitalisation has extended semiotics “to the core of objectivity” (Latour, 2008). Indeed, new parents have been described as “low-hanging fruit for gadget-makers because they want to give their babies the best start in life” (Kleinman, 2017).

Meanwhile, ethnographic analyses of emergency medicine reveal that patients are often called on to justify the legitimacy of their need, and claim to treatment, through appropriate forms of self-presentation, subject to unspoken medical and moral criteria (Hillman, 2014). In the antenatal clinic, women also become ‘particular ethical subjects’ (Johnson, 2014). By providing new means of self-expression, self-report technologies may therefore increase the pressure on women to present themselves according to the ‘correct rules’ of patient behaviour and the patient-typifications on which medical decisions are based.

Designers of personal and public information technologies must navigate these tensions of personal and collective wellbeing, while attending to those practices which our choices serve to normalise. The tools we create progressively realise medical practice, individual responsibility and normative ethics. If, as Latour suggests, technology is ‘society made durable’ (Latour, 1990) then addressing tensions of personal information and public health requires a design practice attentive to the inscription of “modes of use” which “inhibit or preclude certain actions while inviting or demanding others” (Akrich and Latour, 1992).

*Strategy 1. Attend to the Framing of Personal Experience within Public Norms*

In previous design research, as in these sessions, clinicians often note that “reference values are needed to make immediate decisions” (Kim et al., 2017). Determining what is normal during pregnancy, however, is made more difficult by the extreme diversity of experience as well as a pervasive stigma surrounding mental health. It is clear from women’s commentary that a fear of adverse consequences features strongly in their thinking regarding honest disclosure. Systematic comparison to the mean also risks presenting mental health and illness in terms of deviance from socio-cultural norms, which may prove counter-productive given evidence of a negative relationship between social comparison and happiness for example (Calvo, Peters, 2014, Mishra, Miller, Haldar, Khelifi, Eschler, Elera, Pollack, Pratt, 2018).

Any representation which frames an individual’s problems as the result of personal inadequacy, or individuality as error, will do little to support disclosure or bridge the mental health treatment gap. Whether we hope to say something about how we ‘should’ live, to ground ourselves in the moment, or to support disclosure, we realise normative ethics through design. It is essential therefore for designers to transparently convey the ways in which a user’s data may be employed, and to work to develop normative practices which support wellbeing.

*Strategy 2. Attend to the Propagation of Epistemic Values*

Self-tracking technologies often embody epistemic values which imply the pursuit of knowledge through numbers, extending to daily life a scientific paradigm which “concerns itself with time-series data rather than immediate experience; correlation rather than causation; patterns rather than events” (Schüll, 2016). Graphing the felt experience of populations and individuals suggests the propagation of these values at scale, potentially turning technological values into societal and individual values.

Perinatal technologies in particular risk turning pregnancy into a scientific enterprise, divorcing women from their subjective experience (Johnson, 2014), problematising the ‘pregnant body,’ and solving this ‘problem’ through medical interventions which “render the pregnant body calculable and manageable” (Gui et al., 2018). As the sole sources of ‘authoritative knowledge,’ systems and services can also then come to suppress “what women might know, think, feel, or imagine about themselves in the childbirth process” (Gui et al., 2018).

To avoid embodying our technologies with a narrow empiricist spirit, we must clearly distinguish the use of a computer science epistemology to seek certain forms of knowledge from the value systems by which we wish to live. The *BrightSelf* interface is designed to mitigate many of the assumptions present in commercial ‘tracking’ technologies, such as paradigms of intrinsically-good tracking, the value of self-discipline, the need for improvement, expectations of self-control and other features of ‘healthism’ (Spiel et al., 2018), by recognising personal, subjective experience and by striving to facilitate support-seeking behaviours among those in need.

*Strategy 3. Support Transparency to Mitigate Misinterpretation*

Participants of these design sessions often spoke of the fear of their data being misinterpreted. To minimise this risk and promote disclosure, designers should seek to support transparency; concerning the limits of what it is possible to reasonably infer from different forms of data, the means by which data shared will be interpreted (by midwives alone, in conversation with women, or through the use of particular sense-making strategies as simple as looking for trends and peaks etc.), and concerning consequences, all possible actions in which data shared might result.

Sharing self-reported data is likely to influence the expectations women hold of a public health service. If carefully set and met, this can support a practice of wellbeing. If not, it can undermine midwives’ and organisational efficacy. Expectations of data use can be managed explicitly and implicitly through considerate design. This might involve imposing limits on communication or access to personal information, or by supporting users’ dignity, including their “fundamental right to be unreachable” (Giráldez and Casal, 2004).

*Strategy 4. Acknowledge Personal and Public Framings of Wellbeing*

These design sessions reflect a need to recognise both personal and public interpretations of what it means to be well. How women are perceived and cared for is a result of both actors, individual midwives’ attitudes and experiences, and structures, those imposed by care pathways and organisational practices, including the data used to represent patients.

The pragmatic detail of interaction design must attend to how labelling and other forms of abstraction shape patient engagement, medical practice and culture. Engaging self-report tools may well support a fuller understanding of the spectrum of emotion and wellbeing in pregnancy. Articulating our experience in terms of dots and lines, however, must not preclude awareness of the constructed and culturally contingent nature of wellbeing.

The design of healthcare technologies takes place in a context where screening for perinatal depression is ideally a means of validating women’s experiences and enabling access to care. Screening has also, however, been associated with patriarchal practices, constraining women to societal expectations of motherhood, denying women the authenticity of a ‘grieving response’ related to multiple losses, powerlessness and status change, and casting aside the socio-cultural and economic contingencies of childbirth in order to grant medical professionals control over women’s bodies (Shaikh and Kauppi, 2015). Designers must maintain awareness of the potential consequences of reductionism and act to avoid dissociation between database and phenomenological selves. As Hillary Putnam writes, “other things being equal, a world in which there are a variety of (morally permissible) conceptions of human flourishing is better than one in which everyone agrees on just one conception” (Putnam, 2002).

The *BrightSelf* interface design pays particular attention to the holistic depiction of women with respect to their data — as bundles of traits and states, as thinking and feeling individuals, and as fixed or changing entities. An array of distinct character icons for example, highlights each client’s individuality, and serves to mitigate a purely reductive form of data interpretation. Our approach to data visualisation is hierarchical, always pointing to the potential for richer forms of understanding in light of an ever more complex spectrum of wellbeing.

## 6 Conclusion

This paper presents insights into the design of technologies for the self-report and disclosure of psychological wellbeing during pregnancy, drawing on the experiences of a diverse group of stakeholders. Public health is a highly complex context for design. Healthcare responsibilities are critical and the volumes in these systems so great that epistemic and technical knowledge often takes precedence for clinicians. Our participants’ comments however, also highlight the need to view activities of self-report, data sharing and feedback not simply as reified acts but as socially-contingent practices with significant implications for care pathways, experiences of care, women’s willingness to interact with services, midwives’ capacity to perform their roles and the public health project as a whole. Many of these findings are also likely to be relevant to other contexts within healthcare where practices of self-report and information sharing take place. We recommend that designers of personal and public health technologies focus on strategies to support negotiation, navigate uncertainty, and realise a shared practice of wellbeing.

Although the tensions highlighted by these sessions could be interpreted as barriers to the design and adoption of health and wellbeing technologies, they are also, in part, what allows these complex ecologies of actors and structures to function. Designers must therefore attend not to the resolution, but to the negotiation of these essential characteristics of antenatal care.

## Declaration of Competing Interest

We wish to confirm that there are no known conflicts of interest associated with this publication and there has been no financial support for this work that could have influenced the outcome.
